# Cancer Drug Approvals That Displaced Existing Standard-of-Care Therapies, 2016-2021

**DOI:** 10.1001/jamanetworkopen.2022.2265

**Published:** 2022-03-15

**Authors:** David J. Benjamin, Alexander Xu, Mark P. Lythgoe, Vinay Prasad

**Affiliations:** 1 Division of Hematology and Oncology, Department of Medicine, University of California, Irvine, Orange; 2 Sidney Kimmel Medical College, Thomas Jefferson University, Philadelphia, Pennsylvania; 3 Department of Surgery and Cancer, Imperial College London, Hammersmith Hospital, London, England; 4 Department of Epidemiology and Biostatistics, University of California, San Francisco

## Abstract

**Importance:**

Although several cancer drugs receive US Food and Drug Administration (FDA) approval each month, it is unclear how many of these cancer drugs transform the treatment landscape significantly by tumor group. Specifically, it remains unclear how many of these newly approved cancer drugs displace the existing standard-of-care therapies for their indication vs being added to existing therapies.

**Objective:**

To examine how many cancer drugs displace the standard-of-care therapies vs being added to existing therapy or filling breaks in systemic treatments in the metastatic setting, adjuvant setting, or maintenance setting.

**Design, Setting, and Participants:**

Retrospective cross-sectional study using landmark trials leading to FDA approval of cancer drugs between May 1, 2016, and May 31, 2021. The study evaluated all FDA approvals for cancer drugs between May 1, 2016, and May 31, 2021, using the FDA Oncology (Cancer)/Hematologic Malignancies Approval Notifications website. All clinical trials leading to FDA approval of cancer drugs during this period were examined.

**Main Outcomes and Measures:**

A drug was determined to have displaced the prior standard-of-care therapy by evaluating the comparator arm (or lack thereof) in the clinical trial leading to the drug’s approval and also by reviewing National Comprehensive Cancer Network Guidelines. Cancer drug approvals were categorized as *first-line displacing* if a drug was approved for use in the first-line setting and displaced the prior standard-of-care drug for an indication, *first-line drug alternatives/new* if a drug was approved for use in the first-line setting but did not displace the standard of care at the time of approval or was a new drug that was first of its class for an approved indication, *add on* if a drug was approved in combination with a previously approved therapy for a disease or if a drug was approved for use in the adjuvant or maintenance settings, and *later line* if a drug was approved for use in the second-, third-, or later-line settings.

**Results:**

Between May 1, 2016, and May 31, 2021, there were 207 FDA cancer drug approvals in oncology and malignant hematology. Of these 207 approvals, 28 drugs (14%) were first-line displacing therapies. A total of 32 drugs (15%) were first-line drug alternatives/new drugs. A total of 61 drugs (29%) were add-on therapies. Finally, 86 drugs (42%) were approved as later-line therapies.

**Conclusions and Relevance:**

In this study, most cancer drug approvals between 2016 and 2021 were in the later-line settings as opposed to displacing the current standard-of-care therapy for the approved indication. These later-line drugs may benefit patients with few alternatives but add to the cost of care because competition in the drug markets is a key factor in leading to lower drug prices.

## Key Points

**Question:**

How many cancer therapies approved by the US Food and Drug Administration between 2016 and 2021 displaced standard-of-care therapies vs being added to existing therapies in the metastatic, adjuvant, and maintenance settings?

**Findings:**

In this cross-sectional study, between May 1, 2016, and May 31, 2021, there were 207 cancer drug approvals in oncology and malignant hematology. Of these therapies, 14% displaced cancer therapies that were previously approved and considered to be the standard of care for their indication; most therapies (42%) were approved as later-line drugs in the second-, third-, or later-line settings.

**Meaning:**

Although there has been a high volume of new cancer drug approvals in oncology and malignant hematology, only a minority of these drug approvals displace the current standard-of-care therapy, suggesting that newer drugs may benefit patients with few alternatives but could add to the cost of care because competition in the drug markets is crucial to lowering drug prices.

## Teaser

This cross-sectional study investigates the number of US Food and Drug Administration–approved cancer drugs that displaced standard-of-care therapies between 2016 and 2021.

## Introduction

The development and approval of new cancer therapies, including immunotherapy, such as the immune checkpoint inhibitors nivolumab and pembrolizumab, and targeted therapies, such as osimertinib and alectinib, during the past decade have led the modern era of cancer treatment to be referred to as the “golden age of oncology.”^1^ In fact, despite the unanticipated obstacles brought on by the COVID-19 global pandemic, the US Food and Drug Administration (FDA) approved more new cancer medicines during 2020 than in the prior year.^2^ These cancer drug approvals have justifiably provided much hope and optimism to patients and the physicians and health care professionals caring for patients with cancer after a drought of several decades without new drug approvals for several cancers.^3,4^

Although several oncology and malignant hematology drugs receive FDA approval each month, it is unclear how many of these cancer drugs transform the treatment landscape significantly for each tumor group. It remains unclear how many of these newly approved cancer drugs displace the existing standard-of-care therapies for their indication compared with providing simply an alternative treatment option. For example, osimertinib first received FDA approval in November 2017 for epidermal growth factor receptor (*EGFR*) T790M variant–positive non–small cell lung cancer (NSCLC) and received supplementary approval in April 2018 as a first-line therapy for NSCLC with epidermal growth factor receptor exon 19 deletion or exon 21 L858R substitution.^5^ Efficacy in the front-line setting was established in the randomized, double-blind, active-controlled FLAURA study,^6^ in which patients were randomized to receive either osimertinib or erlotinib/gefitinib. The osimertinib arm showed significant superiority over erlotinib/gefitinib, therefore displacing these drugs to become the treatment of choice for this indication.

Conversely, avelumab was approved as maintenance therapy for locally advanced or metastatic urothelial carcinoma after first-line platinum-containing chemotherapy. The JAVELIN Bladder 100 study was a randomized phase 3 trial that assigned patients with unresectable locally advanced or metastatic urothelial cancer who did not progress while taking first-line chemotherapy to receive either maintenance immune checkpoint inhibitor avelumab or best supportive care.^7^ The study demonstrated that maintenance avelumab prolonged overall survival (21.4 months; 95% CI, 18.9-26.1) compared with best supportive care (14.3 months; 95% CI, 12.9-17.9).^7^ Following the results of JAVELIN Bladder 100, the standard of care for patients with urothelial cancer who do not progress while taking first-line chemotherapy is maintenance avelumab.

Competition is one of the most critical factors in the drug markets that leads to lower drug prices.^8^ Therefore, it is important to evaluate among cancer drug approvals what percentage of drugs are approved for use in settings that lead to increased competition, and ultimately the possibility of reduced drug prices, as opposed to cancer drugs that prolong survival but do not necessarily compete against each other in the market. The literature is sparse regarding analysis of cancer drug approvals that are categorized according to the potential to promote competition and ultimately lead to lower drug prices.

Given the lack of data on oncology and malignant hematology drug approvals and their positions in the treatment landscape for their indications, we sought to examine how many newly approved cancer drugs displace the standard-of-care therapies compared with becoming an alternative option in the metastatic, adjuvant setting, or maintenance setting. We also wished to evaluate how many new therapies are combined with older cancer therapies.

## Methods

We reviewed the FDA Oncology (Cancer)/Hematologic Malignancies Approval Notifications website^9^ to evaluate all malignant hematology and oncology drugs that were approved between May 1, 2016, and May 31, 2021. Using the FDA approval notices and National Comprehensive Cancer Network Guidelines, the 4 authors evaluated all malignant hematology and oncology drug approvals to determine which newly approved drugs displaced the existing standard-of-care drugs, which were used in combination with previously approved drugs, which were approved for use in the adjuvant or maintenance settings, and which were approved for use in the second-, third-, or later-line settings.^10^ Per policies at the University of California, Thomas Jefferson University, and Imperial College London, the study was not submitted for institutional review board approval because all data are publicly available and because the study did not involve personal health care information. This report followed the Strengthening the Reporting of Observational Studies in Epidemiology (STROBE) reporting guideline for cross-sectional studies.

### Data Analysis

Data for this cross-sectional study were collected and analyzed using Excel, version 16.2 (Microsoft Corporation). Clinical trials deemed “pivotal” for drug approval were analyzed by 2 researchers independently (A.X. and M.P.L.), with any conflicting results being resolved by consensus from a third researcher (D.J.B.). Therapies were only deemed to be displacing if they were approved on the basis of improved efficacy, such as overall survival, progression-free survival, or another surrogate end point deemed suitable by the FDA for approval over established standard-of-care therapy options in this setting. Therapies that were not directly compared, such as in a single-arm study, or that were not compared with established standard-of-care options were considered to be alternative options rather than displacing therapies. Therapies that were added to existing, established treatment options, such as pembrolizumab with cytotoxic chemotherapy in NSCLC in KEYNOTE-189,^18^ were deemed to be add-on therapies rather than displacing therapies. These categorizations were also validated with the National Comprehensive Cancer Network guidelines to ensure validity of therapy options at the date of censoring.^10^

We coded approvals by the following categories: *first-line displacing* if a drug was approved for use in the first-line setting and displaced the prior standard-of-care drug for an indication, *first-line drug alternatives/new* if a drug was approved for use in the first-line setting but did not displace the standard of care at the time of approval or was a new drug that was first of its class for an approved indication, *add on* if a drug was approved in combination with a previously approved therapy for a disease or if a drug was approved for use in the adjuvant or maintenance settings, and *later line* if a drug was approved for use in the second-, third-, or later-line settings. Later-line drugs are therapies that do not necessarily compete against each other in the market and therefore cannot potentially lead to lower drug prices, whereas *first-line drug alternatives/new* promote competition against standard-of-care therapies in the drug market. Descriptive analyses of the collected data were then conducted. No statistical analysis was conducted owing to the nature of the study.

## Results

**Figure 1.**
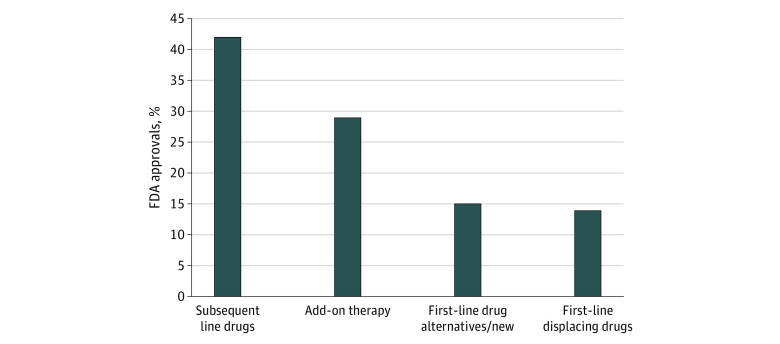
Percentage of US Food and Drug Administration (FDA) Approvals Between May 1, 2016, and May 31, 2021, by Setting of Therapy

There were 207 FDA cancer drug approvals in oncology and malignant hematology between May 1, 2016, and May 31, 2021. Of these 207 approvals, 28 drugs (14%) were first-line therapies that displaced cancer therapies previously approved and considered to be the standard of care for their indication, as indicated by Figure 1. Thirty-two drugs (15%) were first-line drug alternatives/new therapies, receiving approvals for treatment in the first-line setting but not displacing the existing standard of care. Sixty-one drugs (29%) were add-on therapies that were approved in addition to previously approved therapies or were approved for use in the maintenance and adjuvant settings. Finally, 86 therapies (42%) were approved as later-line drugs for use in the second-, third-, or later-line settings.

Examples of the cancer drugs that displaced previously approved therapies include alectinib for anaplastic lymphoma kinase rearrangement–positive metastatic NSCLC, osimertinib for epidermal growth factor receptor exon 19 deletion or exon 21 L858R substitution NSCLC, atezolizumab and bevacizumab for unresectable or metastatic hepatocellular carcinoma, and cabozantinib for advanced kidney cancer. A complete list of first-line displacing drugs is provided in the eAppendix in the Supplement. The ALEX trial^11^ studied alectinib vs crizotinib in patients with previously untreated, advanced anaplastic lymphoma kinase rearrangement–positive NSCLC. The randomized, open-label, phase 3 study demonstrated superior efficacy and lower toxicity with alectinib compared with crizotinib, thus leading to alectinib becoming the first-line therapy for anaplastic lymphoma kinase rearrangement–positive NSCLC per National Comprehensive Cancer Network guidelines and displacing all other anaplastic lymphoma kinase inhibitors at the time of its approval. The IMbrave150 study^12^ evaluated the combination of atezolizumab and bevacizumab in the setting of unresectable or metastatic hepatocellular carcinoma and demonstrated improved overall and progression-free survival in the combination therapy group compared with sorafenib. At the time of this publication, atezolizumab and bevacizumab remain the first-line preferred therapy for unresectable or metastatic unresectable or metastatic hepatocellular carcinoma with Child-Pugh A cirrhosis. Finally, the CABOSUN trial^13^ evaluated the tyrosine kinase inhibitor cabozantinib vs sunitinib as initial therapy for metastatic kidney cancer. The randomized phase 2 trial demonstrated improved progression-free survival with cabozantinib compared with sunitinib.^13^ At the time of its approval, cabozantinib was considered a first-line therapy for metastatic kidney cancer because it had displaced the prior standard-of-care drug sunitinib.^14^

Several cancer drug approvals were in the first-line setting but did not displace the standard-of-care therapy at the time of approval; rather, these drugs were first-line alternative therapies or first-of-their-class therapies. Examples of these drug approvals include apalutamide for nonmetastatic castrate-resistant prostate cancer, tepotinib for metastatic *MET* exon 14–skipping NSCLC, and avapritinib for unresectable or metastatic gastrointestinal stromal tumor (GIST) with platelet-derived growth factor receptor alpha (*PDGFRA*) exon 18 variant including D842V variant. A complete list of approvals in the first-line setting that are first-line alternatives or first-of-their-class therapies is available in the eAppendix in the Supplement. Apalutamide was studied in the randomized, double-blind, phase 3 TITAN study comparing androgen-deprivation therapy in combination with either apalutamide or placebo.^15^ The study revealed improved overall survival at 24 months (82.4% in the apalutamide group vs 73.5% in the placebo group) as well as improved radiographic progression-free survival at 24 months (68.2% in the apalutamide group vs 47.5% in the placebo group). Although apalutamide is approved for nonmetastatic castrate-resistant prostate cancer, it is considered a first-line treatment that is an alternative to darolutamide or enzalutamide depending on clinician preference and a patient’s comorbidities, among other factors.

In February 2021, the FDA granted accelerated approval to tepotinib for *MET* exon 14–skipping NSCLC based off results from the VISION trial,^16^ an open-label phase 2 study. The study showed that approximately half of the enrolled patients responded to tepotinib. Tepotinib is considered to be a first-line alternative to capmatinib, which is similarly approved for *MET* exon 14–skipping NSCLC. Because the VISION trial did not compare these 2 tyrosine kinase inhibitors, there is no clear preferred first-line therapy for *MET* exon 14–skipping NSCLC. Avapritinib, a tyrosine kinase inhibitor, was studied in the NAVIGATOR trial, which was a single-arm, open-label study evaluating efficacy in patients with unresectable GIST including those with *PDGFRA* D842V variant–positive GIST. Among patients with *PDGFRA* D842V variants, the overall response rate was 91%, with the median duration of response being 27.6 months (95% CI, 17.6 to not reached).^17^ The study subsequently led to the approval of avapritinib for patients with *PDGFRA* D842V variant–positive GIST as the first-of-class therapy for GIST harboring a *PDGFRA* exon 18 variant.

Eighty-six (42% of all cancer drug approvals between May 1, 2016, and May 31, 2021) cancer drugs were approved for use “in combination” with previously approved therapies or approved for use in the adjuvant, consolidation, or maintenance settings and effectively are add-on therapies. For example, among oncology drug approvals, pembrolizumab was approved in combination with chemotherapy (with carboplatin and pemetrexed for nonsquamous histological characteristics based on data from KEYNOTE-189^18^ and with carboplatin and paclitaxel for squamous NSCLC based on data from KEYNOTE-407^19^) for the indication of advanced NSCLC. The chemotherapy regimens used in the treatment of nonsquamous NSCLC and squamous NSCLC remained unchanged following these 2 landmark studies, KEYNOTE-189^18^ and KEYNOTE-407,^19^ except for the addition of the immune checkpoint inhibitor pembrolizumab. Another example of a new malignant hematology drug that was approved for use in combination with previously approved therapies is venetoclax, which was approved in combination with hypomethylating agents (HMAs) in adults aged 75 years or older with newly diagnosed acute myeloid leukemia and who are not candidates for intensive induction chemotherapy. Venetoclax, a B-cell lymphoma-2 inhibitor, was studied in combination with HMAs azacitadine and decitabine in treatment-naive older patients with acute myeloid leukemia. The landmark study evaluating venetoclax in combination with HMAs found 67% of patients (n = 145) achieved complete remission.^20^ Because HMAs had previously been used in the treatment of frail older patients, venetoclax was approved to be used as an add-on therapy with these HMAs in this patient population with untreated acute myeloid leukemia.

With regard to examples of maintenance therapy that we categorized as add-on therapy to induction or primary treatment, durvalumab was approved as maintenance therapy for unresectable stage III NSCLC without progression after concurrent platinum-based chemotherapy and radiation therapy. The PACIFIC trial^21^ studied the immune checkpoint inhibitor durvalumab in the setting of patients with stage III NSCLC who did not have disease progression after 2 or more cycles of platinum-based chemoradiotherapy. The randomized, phase 3 trial found that the median progression-free survival was higher in patients with durvalumab (16.8 months; 95% CI, 13.0-18.1) compared with patients who received placebo (5.6 months; 95% CI, 4.6-7.8).^21^ At the time of this publication, the standard of care for patients with stage III NSCLC who do not have disease progression after chemoradiotherapy (and with no contraindications to immunotherapy) is now up to 12 months of consolidation durvalumab.

Most cancer drug approvals (n = 86) were in the later-line settings. Examples of drugs that were approved for the later-line setting include copanlisib, which is approved for patients with relapsed follicular lymphoma who received 2 prior therapies, and moxetumomab pasudotox-tdfk, which is approved for patients with relapsed or refractory hairy cell leukemia who received 2 prior systemic therapies. A phase 2 study evaluated PI3K inhibitor copanlisib^22^ in the setting of relapsed or refractory indolent or aggressive lymphoma. The study included 33 patients with indolent lymphoma and 51 patients with aggressive lymphoma, with follicular lymphoma (48.5%) being the most common histological subtype. The objective response rate among patients in the indolent lymphoma cohort was 43.7% (n = 14 of 32). The study was conducted with a single arm and therefore did not compare copanlisib with any other therapies. Copanlisib was subsequently approved for use in the third-line setting and remains a third-line therapy for relapsed or refractory follicular lymphoma.^22^ Moxetumomab pasudotox-tdfk, a recombinant CD22-directed cytotoxin, was studied in a single-arm phase 3 trial^23^ in patients with relapsed or refractory hairy cell leukemia with at least 2 or more prior systemic therapies. The overall complete remission rate was 41.3% in this heavily pretreated patient cohort.^23^ Moxetumomab pasudotox-tdfk was approved for use in the third line for hairy cell leukemia and remains a third-line therapy option. Most second-, third-, or later-line therapies were studied in single-arm studies or vs placebo and therefore did not displace any therapies because of their approvals.

**Figure 2.**
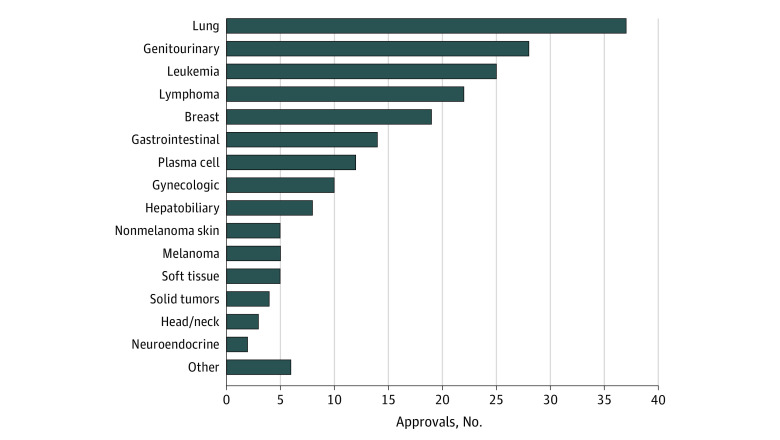
Number of Oncologic Approvals Between May 1, 2016, and May 31, 2021, by Tumor Organ System

Of note, there were disparities among the drug approvals depending on tumor type. Lung-related tumors received the most number of approvals (n = 37), followed by genitourinary tumors (n = 28), leukemia (n = 25), lymphoma (n = 22), breast cancer (n = 19), gastrointestinal cancers (n = 14), plasma cell dyscrasias (n = 12), gynecologic cancers (n = 10), hepatobiliary tumors (n = 8), nonmelanoma skin cancer (n = 5), melanoma (n = 5), soft-tissue cancers (n = 5), tumor agnostic (n = 4), head and neck cancers (n = 3), and neuroendocrine cancer (n = 2), as shown in Figure 2. Several cancer types had only 1 drug approval between May 1, 2016, and May 31, 2021, and did not fall into any of any of these categories. These included anaplastic thyroid cancer, myelodysplastic syndrome, mesothelioma, tenosynovial giant cell tumor, Erdheim-Chester disease, and blastic plasmacytoid dendritic cell neoplasm. A comprehensive list of approvals by tumor type is provided in the eAppendix in the Supplement.

## Discussion

In this cross-sectional study, between May 1, 2016, and May 31, 2021, most of the 207 FDA drug approvals for oncology and malignant hematology drugs were for therapies to be used in the second-, third-, or later-line setting, with 86 drug approvals (42%) falling in this category. Approximately 1 in 6 newly approved drugs (28 drugs; 14% of approvals) displaced the therapies that were considered to be the standard of care for their indication at the time of approval, while 32 drugs were approved for use in the first-line setting as alternative first-line options or new first-of-their-class drugs. Both of these categories may provide market competition and work to lower cancer drug prices; however, practical examples of competition on the basis of price are rare in oncology.^24^ The remaining 61 drug approvals (29%) came in the adjuvant or maintenance settings used in combination with previously approved therapies.

Four new approvals (2%) were in the tumor agnostic setting, including neurotrophic tyrosine receptor kinase inhibitors entrectinib and larotrectinib, both approved for patients with neurotrophic tyrosine receptor kinase variants who have no satisfactory alternative treatments, and 2 approvals for the anti–programmed cell death 1 drug pembrolizumab in cancers when patients have no satisfactory alternative treatment options and have either a high tumor variant burden or have high levels of microsatellite instability or mismatch repair deficiency. These new therapies did not displace any existing therapies, thereby becoming viable new therapies in the second line and beyond.

While there is justified enthusiasm for the high volume of new cancer drug approvals in oncology and malignant hematology, these approvals must be evaluated in the context of their use (first-line therapies, with some altering the treatment paradigm for a certain indication, as opposed to third- or fourth-line options for patients). It is evident that further research and cancer drug development is required to drastically change the treatment landscape for many oncologic and malignant hematologic conditions.

### Limitations

This study has several limitations. Some drugs may displace later lines of therapy, although we did not include them in this study. For example, avelumab used as first-line maintenance therapy in urothelial cancer may displace second-line immune checkpoint inhibitor use. Therapies may also displace alternative treatment modalities; for example, radium-223 in prostate cancer may displace external beam radiotherapy but was deemed beyond the scope of this study. In addition, the potential number of patients affected by—and, by extension, the market impact—from each drug approval varies considerably. However, this analysis was deemed beyond the scope of this study.

## Conclusions

This study found that 14% of new drug approvals displaced existing standards of care, and an additional 15% provided market competition. At the same time, 29% were add-on or maintenance drugs that can only increase the cost of care. Forty-two percent were drugs approved for patients who had exhausted other treatment options. These later-line drugs may benefit patients with few alternatives but also add to cost of care and further delay palliative and comfort services. Further incentives to maximize competition in the cancer drug space warrant consideration.

## Notes

Supplement. **eAppendix.** Drug Approvals in Oncology that “Displace” Currently Approved Drugs

## References

[1] Landau D. Why advances in immunotherapy mean the golden age of oncology. Oncol Times. 2019;41(13):24, 32. doi:10.1097/01.COT.0000574936.15457.93

[2] Smith CEP, Prasad V. Assessment of new molecular entities approved for cancer treatment in 2020. JAMA Netw Open. 2021;4(5):e2112558. doi:10.1001/jamanetworkopen.2021.12558 34047795 PMC8164099

[3] Davis JR, Benjamin DJ, Jonas BA. New and emerging therapies for acute myeloid leukaemia. J Investig Med. 2018;66(8):1088-1095. doi:10.1136/jim-2018-000807 30127098 PMC6733983

[4] Suzman DL, Agrawal S, Ning YM, . FDA approval summary: atezolizumab or pembrolizumab for the treatment of patients with advanced urothelial carcinoma ineligible for cisplatin-containing chemotherapy. Oncologist. 2019;24(4):563-569. doi:10.1634/theoncologist.2018-0084 30541754 PMC6459239

[5] Greig SL. Osimertinib: first global approval. Drugs. 2016;76(2):263-273. doi:10.1007/s40265-015-0533-4 26729184

[6] Soria JC, Ohe Y, Vansteenkiste J, ; FLAURA Investigators. Osimertinib in untreated *EGFR*-mutated advanced non-small-cell lung cancer. N Engl J Med. 2018;378(2):113-125. doi:10.1056/NEJMoa1713137 29151359

[7] Powles T, Park SH, Voog E, . Avelumab maintenance therapy for advanced or metastatic urothelial carcinoma. N Engl J Med. 2020;383(13):1218-1230. doi:10.1056/NEJMoa2002788 32945632

[8] Sarpatwari A, DiBello J, Zakarian M, Najafzadeh M, Kesselheim AS. Competition and price among brand-name drugs in the same class: a systematic review of the evidence. PLoS Med. 2019;16(7):e1002872. doi:10.1371/journal.pmed.1002872 31361747 PMC6667132

[9] US Food and Drug Administration. Oncology (cancer) / hematologic malignancies approval notifications. July 16, 2021. Accessed July 16, 2021. https://www.fda.gov/drugs/resources-information-approved-drugs/oncology-cancer-hematologic-malignancies-approval-notifications

[10] NCCN. Treatment by cancer type. Accessed July 16, 2021. https://www.nccn.org/guidelines/category_1

[11] Peters S, Camidge DR, Shaw AT, ; ALEX Trial Investigators. Alectinib versus crizotinib in untreated ALK-positive non-small-cell lung cancer. N Engl J Med. 2017;377(9):829-838. doi:10.1056/NEJMoa1704795 28586279

[12] Finn RS, Qin S, Ikeda M, ; IMbrave150 Investigators. Atezolizumab plus bevacizumab in unresectable hepatocellular carcinoma. N Engl J Med. 2020;382(20):1894-1905. doi:10.1056/NEJMoa1915745 32402160

[13] Choueiri TK, Hessel C, Halabi S, . Cabozantinib versus sunitinib as initial therapy for metastatic renal cell carcinoma of intermediate or poor risk (alliance A031203 CABOSUN randomised trial): progression-free survival by independent review and overall survival update. Eur J Cancer. 2018;94:115-125. doi:10.1016/j.ejca.2018.02.012 29550566 PMC6057479

[14] Lyseng-Williamson KA. Cabozantinib as first-line treatment in advanced renal cell carcinoma: a profile of its use. Drugs Ther Perspect. 2018;34(10):457-465. doi:10.1007/s40267-018-0547-6 30679901 PMC6323107

[15] Chi KN, Agarwal N, Bjartell A, ; TITAN Investigators. Apalutamide for metastatic, castration-sensitive prostate cancer. N Engl J Med. 2019;381(1):13-24. doi:10.1056/NEJMoa1903307 31150574

[16] Paik PK, Felip E, Veillon R, . Tepotinib in non-small-cell lung cancer with *MET* exon 14 skipping mutations. N Engl J Med. 2020;383(10):931-943. doi:10.1056/NEJMoa2004407 32469185 PMC8422679

[17] Heinrich MC, Jones RL, von Mehren M, et al. Avapritinib in advanced *PDGFRA* D842V-mutant gastrointestinal stromal tumour (NAVIGATOR): a multicentre, open-label, phase 1 trial. *Lancet Oncol*. 2020;21(7):935-946. doi:10.1016/S1470-2045(20)30269-2 32615108

[18] Gandhi L, Rodríguez-Abreu D, Gadgeel S, ; KEYNOTE-189 Investigators. Pembrolizumab plus chemotherapy in metastatic non-small-cell lung cancer. N Engl J Med. 2018;378(22):2078-2092. doi:10.1056/NEJMoa1801005 29658856

[19] Paz-Ares L, Luft A, Vicente D, ; KEYNOTE-407 Investigators. Pembrolizumab plus chemotherapy for squamous non-small-cell lung cancer. N Engl J Med. 2018;379(21):2040-2051. doi:10.1056/NEJMoa1810865 30280635

[20] DiNardo CD, Pratz K, Pullarkat V, . Venetoclax combined with decitabine or azacitidine in treatment-naive, elderly patients with acute myeloid leukemia. Blood. 2019;133(1):7-17. doi:10.1182/blood-2018-08-868752 30361262 PMC6318429

[21] Antonia SJ, Villegas A, Daniel D, ; PACIFIC Investigators. Durvalumab after chemoradiotherapy in stage III non-small-cell lung cancer. N Engl J Med. 2017;377(20):1919-1929. doi:10.1056/NEJMoa1709937 28885881

[22] Dreyling M, Morschhauser F, Bouabdallah K, . Phase II study of copanlisib, a PI3K inhibitor, in relapsed or refractory, indolent or aggressive lymphoma. Ann Oncol. 2017;28(9):2169-2178. doi:10.1093/annonc/mdx289 28633365 PMC5834070

[23] Kreitman RJ, Dearden CE, Zinzani PLL, . Moxetumomab pasudotox-tdfk in heavily pretreated patients with relapsed/refractory hairy cell leukemia (HCL): long-term follow-up from the pivotal phase 3 trial. Blood. 2019;134(suppl_1):2808. doi:10.1182/blood-2019-122307

[24] Prasad V, De Jesús K, Mailankody S. The high price of anticancer drugs: origins, implications, barriers, solutions. Nat Rev Clin Oncol. 2017;14(6):381-390. doi:10.1038/nrclinonc.2017.31 28290490

